# Development of novel antibodies for detection of mobile colistin-resistant bacteria contaminated in meats

**DOI:** 10.1038/s41598-018-34764-2

**Published:** 2018-11-13

**Authors:** Xiaohua He, Daniela Mavrici, Stephanie Patfield, Fernando M. Rubio

**Affiliations:** 1 Western Regional Research Center, U.S. Department of Agriculture, Agricultural Research Service, Albany, 94710 USA; 2 Present Address: Plexxikon Inc., 91 Bolivar Drive, Berkeley, 94710 USA; 3 Abraxis, Inc., Warminster, 18974 USA

## Abstract

The recent discovery and rapid spread of mobile colistin-resistant gene, *mcr*-1, among bacteria isolated from a broad range of sources is undermining our ability to treat bacterial infections and threatening human health and safety. To prevent further transfer of colistin resistance, practical and reliable methods for *mcr*-1-containing bacteria are need. In this study, standards and novel polyclonal and monoclonal antibodies (mAbs) against MCR-1 were developed. Among nine mAbs, three were MCR-1 specific and six cross-reacted with both MCR-1 and MCR-2. A sandwich enzyme-linked immunosorbent assay (ELISA) was established using the polyclonal antibody as a capturer and the mAb MCR-1-7 as a detector. The assay had a limit of detection of 0.01 ng/mL for MCR-1 and 0.1 ng/mL for MCR-2 in buffer with coefficients of variation (CV) less than 15%. When applied to ground beef, chicken and pork, this ELISA identified samples inoculated with less than 0.4 cfu/g of meat, demonstrating its strong tolerance to complex food matrices. To our knowledge, this is the first immunoassay developed for MCR-1 and MCR-2. It should be useful for prompt and reliable screening of meat samples contaminated with plasmid-borne colistin-resistant bacteria, thus reducing human risk of foodborne infections with possibly no antibiotic treatment options.

## Introduction

Colistin, also known as polymyxin E, is a bactericidal antibiotic with a broad Gram-negative spectrum. It acts by binding to the anionic lipopolysaccharide (LPS) and phospholipids in the outer cell membrane of bacteria, displacing divalent cations from the phosphate groups of membrane lipids. Bacteria are killed by the ensuring disruption of the outer cell membrane and leakage of intracellular contents^1^. Formerly, colistin resistance was thought to be solely caused by chromosomal mutations in genes involved in synthesis or modification of the lipid A, resulting in inefficient binding of polymyxins to the lipid A on the LPS^2^. Chromosomal colistin resistance may cause clonal spread in clinical settings^3^, but usually does not spread via horizontal gene transfer, thus has limited impact to outbreaks. However, in November 2015, a Chinese group discovered several colistin-resistant bacterial strains isolated from food animals and humans harboring a plasmid-borne colistin-resistant gene, *mcr*-1. This gene can spread from one type of bacteria to another^4^. It encodes a phosphoethanolamine (PEA) transferase that catalyzes the addition of cationic PEA to lipid A component of LPS, reducing the negative charge of the cell surface such that the cationic colistin is unable to bind and initiate membrane lysis^5^. This was the first report for a transmissible colistin resistance determinant. Since then, plasmid-borne *mcr*-1 has been found in bacteria isolated from various food animals, the environment, vegetables and patients and spread onto at least five continents and more than 40 countries^6–8^. This resistance mechanism has even extended to carbapenemase-producing Enterobacteriaceae that are resistant to the carbapenem class of antibiotics, considered the drugs of last resort for such infections^9^. These discoveries are of great concern. Although colistin is an old drug that was discontinued for routine use in humans due to its kidney toxicity^1^, increased antimicrobial resistance, particularly the emergence of carbapenem resistance in clinically important Gram-negative bacteria, has renewed interest in colistin as a therapeutic option^10,11^. Recently, new plasmid-mediated colistin-resistant genes, *mcr*-2 to *mcr*-8 were identified^12–18^ and PCR-based screening found an even higher prevalence of *mcr*-2 than *mcr*-1 in *Escherichia coli* (*E*. *coli*) strains isolated from porcine and bovine^12^. It was found that *mcr*-2 to *mcr*-8 genes have 44 to 77% nucleotide identity to *mcr*-1 and their gene products have 32 to 83% amino acid sequence identity to MCR-1. The identity levels of the N-terminal transmembrane domain and the C-terminal catalytic domain are 72% and 87.4% between MCR-1 and MCR-2. Both MCR-1 and MCR-2 account for bacterial resistance to colistin through catalyzing chemical modification of the LPS-lipid A moiety with addition of phosphoethanolamine to the phosphate group at the 4′ position of the sugar^19^.

To prevent further spread of mobile colistin resistance to hospitals and other healthcare settings, which has already been observed^4^, timely screening for *mcr*-positive bacteria is critical. Currently, the main detection methods for MCR-producing bacteria can be classified into phenotypic and genotypic methods. Phenotypic methods are mainly culture-based antibiotic sensitivity testing by estimation of MIC using broth microdilution (BMD). These methods are cheap and easy to use, but have a long turnaround time and high discrepancies in MICs due to the polycationic nature of colistin that results in loss under experimental conditions^20–22^. Genotypic methods include conventional PCR, real-time PCR and whole genome sequencing (WGS). PCR methods were used to amplify *mcr* genes mediating resistance to colistin^23–25^. These methods are faster than the phenotypic tests without an initial culturing step. However, PCR provides only indirect evidence for the presence of the *mcr* gene product, MCR protein, as it only targets the encoding nucleic acid. It often has a high frequency of false-negative or unclear results due to the small sample volume and inhibitors present in biological samples^26^, and a high frequency of false-positive results due to cryptic target sequences^27,28^, or dysfunction of promoters and operons responsible for colistin expression. It has been reported that a *mcr*-1 fragment was amplified by PCR from a colistin-susceptible *E*. *coli* strain from the fecal sample of a healthy pig^29^. WGS was used to understand the genetic determinants of resistance to colistin beyond the confines of the identified genes, followed by comparative mapping to a susceptible reference strain’s genome to identify new resistance determinants^4^. However, this method requires skills and expensive equipment and reagents that are not commonly available in most laboratories in developing countries. To increase the current capability of detecting colistin-resistant bacteria, we sought to develop immunoassays that directly detect the presence or absence of the MCR protein, which is the functional enzyme responsible for colistin-resistance.

Here we report the development of MCR-1 and MCR-2 standards, novel polyclonal and monoclonal antibodies (mAbs) against the MCR-1 and MCR-2 proteins and demonstrate the performance of these antibodies in various applications. A sandwich enzyme-linked immunosorbent assay (ELISA) was established and comprehensively evaluated with MCR-1 and MCR-2 standards, bacterial cultures, and simulated cross-contamination of meat samples. Our results indicate that the ELISA was highly sensitive, specific and has strong tolerance to complex food matrices, such as ground beef, chicken and pork, which is extremely valuable because colistin is widely used for controlling diarrheal diseases in cow, pig and poultry production^30^, and high prevalence of colistin-resistant bacteria has already been found in these food animals^6,31,32^.

## Results and Discussion

### Generation of recombinant MCR-1 and MCR-2 proteins

**Figure 1:**
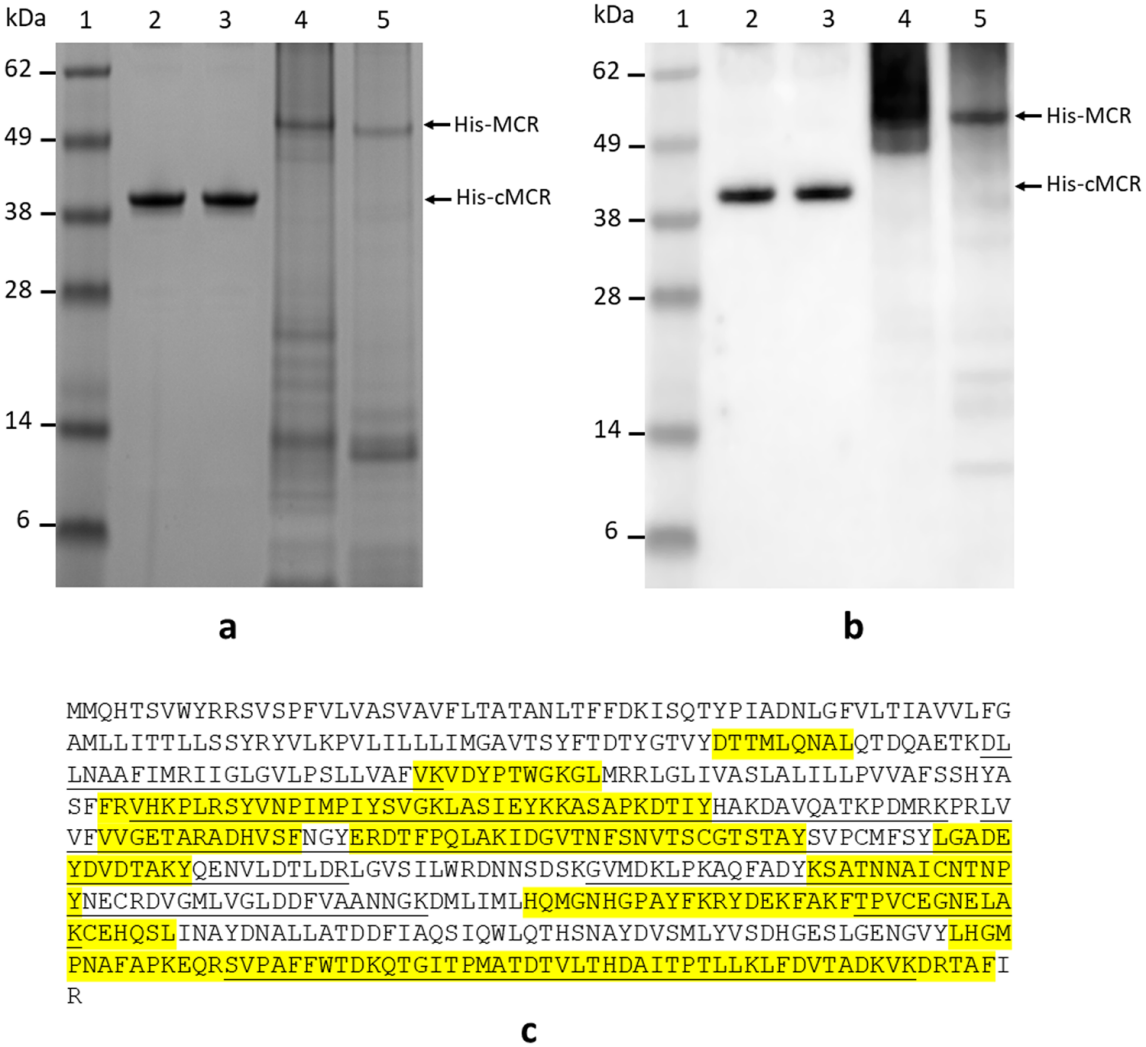
Analysis of His-tagged recombinant MCR-1 and MCR-2 proteins. (**a**) Cropped SDS-PAGE of partially purified MCR recombinant proteins stained with SimplyBlue: lane 1, Protein markers with molecular weights (kDa) indicated at the left; lane 2, cMCR-1; lane 3, cMCR-2; lane 4, MCR-1; lane 5, MCR-2. Full-length gel is presented in Supplementary Fig. S1a. (**b**) Cropped Western blot of partially purified MCR recombinant proteins using anti-His antibody: lane 1, Protein markers with molecular weights (kDa) indicated at the left; lane 2, cMCR-1; lane 3, cMCR-2; lane 4, MCR-1; lane 5, MCR-2. The predicted His-MCR (full-length) and His-cMCR (catalytic domain) protein bands are indicated by arrows at the right. Full-length blot is presented in Supplementary Fig. S1b. (**c**) MS verification of the N-terminal His-tagged MCR-1 full-length protein. The tryptic peptides (underlined) and the chymotryptic peptides (highlighted in yellow) cover 48.6% and 42.5% of the MCR-1 sequence, respectively. The overall coverage from tryptic and chymotryptic peptides was 60.6%.

Full-length recombinant MCR-1 and MCR-2 proteins with an N-terminal His_6_-tag sequence were expressed in *E*. *coli* BL21 DE3 cells and purified using a HisTrap FF column (see Methods). The predicted size of the His_6_-MCR-1 and His_6_-MCR-2 is ~61 kDa based on GenBank sequences (AKF16168.1 and SBV31106.1), but the size revealed on SDS-PAGE and western blots looked smaller than 61 kDa (Fig. 1a,b, lanes 4 and 5). These proteins are highly insoluble because they consist of five transmembrane domains in their N-terminal 200 amino acids and very difficult to purify. To confirm the intactness of the proteins we produced, mass spectrometry (MS) was performed. The N-terminal His-tagged full-length MCR-1 was digested with trypsin and chymotrypsin. The peptides obtained from two proteases covered 60.6% of the MCR-1 sequence (Fig. 1c). This suggests that the intact MCR-1 was recovered from *E*. *coli*. The failure to identify the N-terminal 100 amino acids by MS might be due to intrinsic hydrophobicity. Liu *et al*. (2016) reported that MCR consists of one membrane-spanning domain (residues 1–214), one soluble catalytic domain (residues 215–541)^4^, and sufficient amounts of soluble catalytic domain have been produced in *E*. *coli*^33^. Therefore, the C-terminal periplasmic catalytic domains of MCR-1 (cMCR-1, residues 214–541) and MCR-2 (cMCR-2, residues 212–538) were produced in *E*. *coli* and purified by HisTrap FF chromatography followed by gel filtration. The resulting protein preparations yielded a major band with molecular weight close to the expected size of His-cMCR, 38.4 kDa (Fig. 1a,b, lanes 2 and 3). Several minor proteins with MW larger than 62 kDa were found in these partially purified protein preparations (Supplementary Fig. 1), the identities of these proteins were unknown. These preparations were used as immunogens and standards for antibody production and immunoassays of MCR-1 and MCR-2 described below.

### Development and characterization of polyclonal and monoclonal antibodies against MCR-1

**Figure 2:**
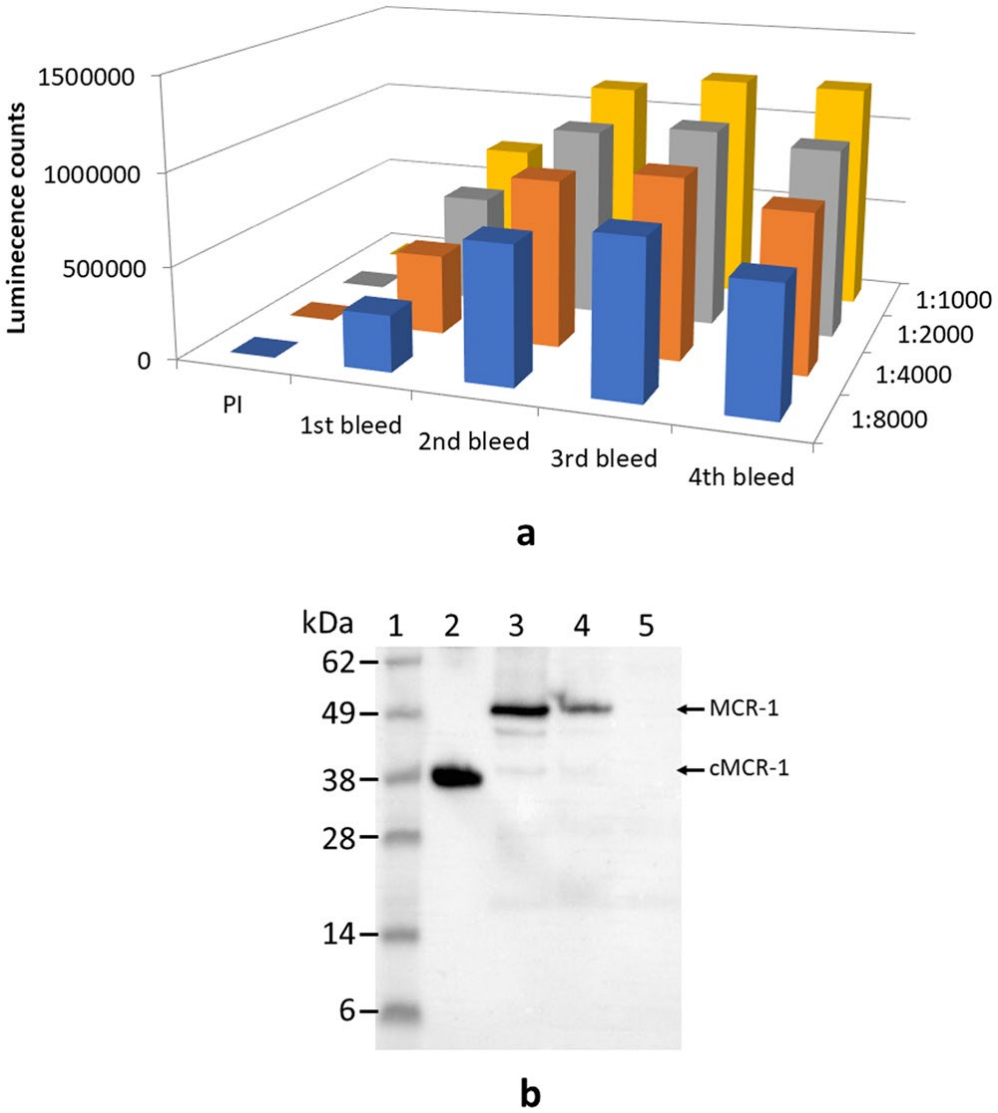
Activity of immunized rabbit serum to MCR-1. (**a**) ELISA analysis of antisera from pre-immune (PI), 1st bleeding, 2nd bleeding, 3rd bleeding, and 4th bleeding. ELISA was performed by directly coating cMCR-1 (10 ng/mL) on the plate, then using antisera of rabbit #1 diluted in a range of 1:1,000–1:8,000 as a detection antibody and goat anti-rabbit-IgG HRP conjugate (200 ng/mL) as a secondary antibody. Each column represents the mean of 3 independent repeats performed in duplicates, the standard deviation for each column ranges from 7 to 51654. (**b**) Cropped Western blot of MCR-1 produced by bacterial cells using immunized rabbit serum IgG. Lane 1, Protein markers with molecular weights (kDa) indicated at the left; Lane 2, Purified cMCR-1 (0.5 µg); Lanes 3 to 5, Cell lysates from bacterial strains AR-Bank #346, AR-Bank #349, and ATCC25922 (colistin negative strain) respectively. Samples were separated by SDS-PAGE under non-reducing condition. The expected sizes of the recombinant MCR-1 catalytic domain and full-length MCR-1 produced by wild type bacteria are indicated at the right side of the blot. Full-length blot is presented in Supplementary Fig. S2b.

**Figure 3:**
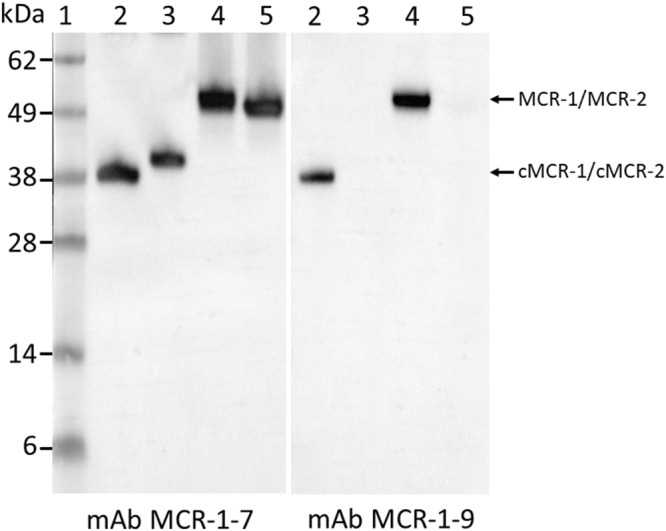
Cropped Western blot of mAb reactivity to MCR-1 and MCR-2. Lane 1, Protein markers with molecular weights (kDa) indicated at the left; Lane 2, Purified cMCR-1 with His-tag removed (0.2 µg); Lane 3, Purified cMCR-2 with His-tag on (0.2 µg); Lanes 4 and 5, Purified His-full-length MCR-1 and His-full-length MCR-2. Blots were probed with indicated mAbs following SDS-PAGE under non-reducing condition. The expected sizes MCR-1 and MCR-2 proteins are indicated at the right side of the blot. Full-length blots are presented in Supplementary Fig. S3.

**Figure 4:**
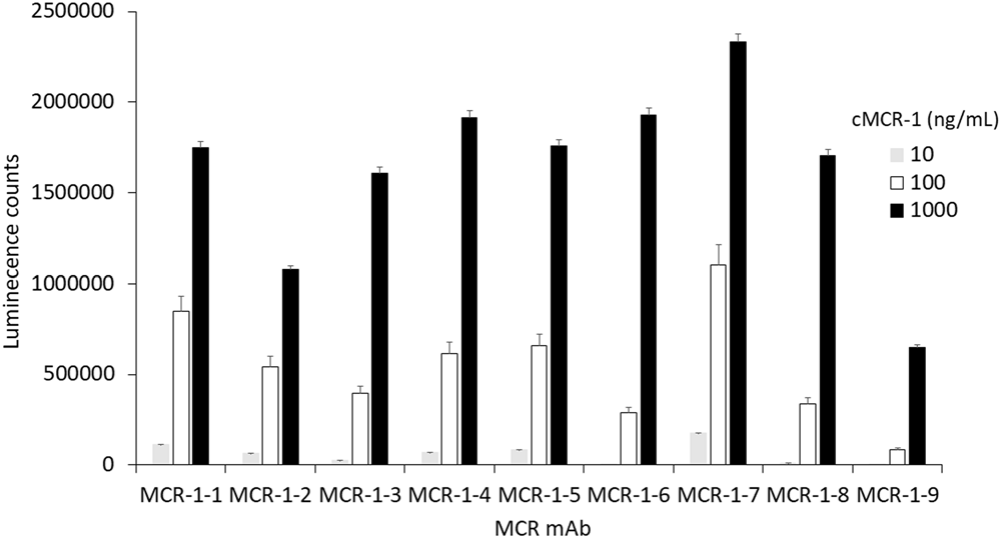
Detection of cMCR-1 by direct ELISA using nine different mAbs. cMCR-1 (10–1,000 ng/mL) was directly coated onto microtiter wells and then incubated with mAbs at 1 µg/mL. Data represent the mean of triplicate plus standard deviation. Three individual experiments were performed.

Antibodies are key to immunoassays and provides the basis for the specificity and sensitivity of the assays. To develop polyclonal and monoclonal antibodies that bind specifically to MCR-1 proteins, the recombinant catalytic domain, cMCR-1 (residues 214–541) with His-tag removed, was used as an immunogen for antibody production (see Methods). Figure 2a shows the ELISA results obtained using one of the immunized rabbit sera. Since the polyclonal antibodies (pAb) made in both rabbits had similar high antibody titers (≥1:8,000) in their sera, the 1^st^, 2^nd^, 3^rd^, and 4^th^ bleed from two rabbits were pooled and used to purify IgG for further experimentation. The IgG purified from the pooled pAb bound to the recombinant cMCR-1 (with His-tag removed) very well. It also bound to a protein with a size close to the full-length recombinant MCR-1 in cell lysates of *mcr*-1 positive strains, AR-Bank #346 and AR-Bank #349, but did not bind anything in cell lysate of *mcr*-1 negative strain, ATCC25922, as demonstrated by Western blot analysis (Fig. 2b). These results suggest that the pAb is specific for the MCR-1. For mAb production and identification, we screened 1000 culture wells following splenocyte-myeloma cell fusion. Nine cell lines were selected for further investigation based on their antibody yields and ELISA titers. The mAbs produced by these cell lines were designated as mAb MCR-1-1 through mAb MCR-1-9. ELISA isotyping study indicated that the mAbs were predominantly IgG1, except for mAb MCR-1-7, which has an IgG2b type heavy chain. All nine mAbs possess kappa light-chains (Table 1). To determine the reactivity of each mAb to MCR-1 and MCR-2, recombinant MCR-1 and MCR-2 catalytic domains and full-length proteins were analyzed by Western blot analyses following SDS-PAGE. Figure 3 demonstrates that mAb MCR-1-7 binds to both MCR-1 and MCR-2, while mAb MCR-1-9 binds only to MCR-1, suggesting that mAb MCR-1-7 recognizes an epitope that is highly homologous in MCR-1 and MCR-2. In contrast, the epitope recognized by mAb MCR-1-9 resides in a region of structural difference between MCR-1 and MCR-2. The specificity of all nine mAbs for MCR-1 and MCR-2 is summarized in Table 1. These antibodies could prove valuable in a variety of applications. The six mAbs cross-reacting with MCR-1 and MCR-2 are important resources for surveillance programs targeting a broad range of colistin-resistant bacteria from human and animals. The three MCR-1 specific mAbs could be useful for source-tracking purposes. The binding affinity of nine mAbs was evaluated by a direct ELISA, in which each mAb was allowed to react with the purified cMCR-1 directly coated onto microplates in phosphate-buffered saline (PBS). Figure 4 demonstrates that the ELISA value for mAb MCR-1-7 is significantly higher than those for the other mAbs (p ≤ 0.01).

**Table 1:** Some characteristics of MCR-1 monoclonal antibodies.

Antibody	Isotype	Specificity
MCR-1-1	IgG1, kappa	MCR-1, MCR-2
MCR-1-2	IgG1, kappa	MCR-1, MCR-2
MCR-1-3	IgG1, kappa	MCR-1, MCR-2
MCR-1-4	IgG1, kappa	MCR-1
MCR-1-5	IgG1, kappa	MCR-1, MCR-2
MCR-1-6	IgG1, kappa	MCR-1
MCR-1-7	IgG2b, kappa	MCR-1, MCR-2
MCR-1-8	IgG1, kappa	MCR-1, MCR-2
MCR-1-9	IgG1, kappa	MCR-1

### Sensitivity, specificity, and linearity of the sandwich ELISA for cMCR-1 and cMCR-2

**Figure 5:**
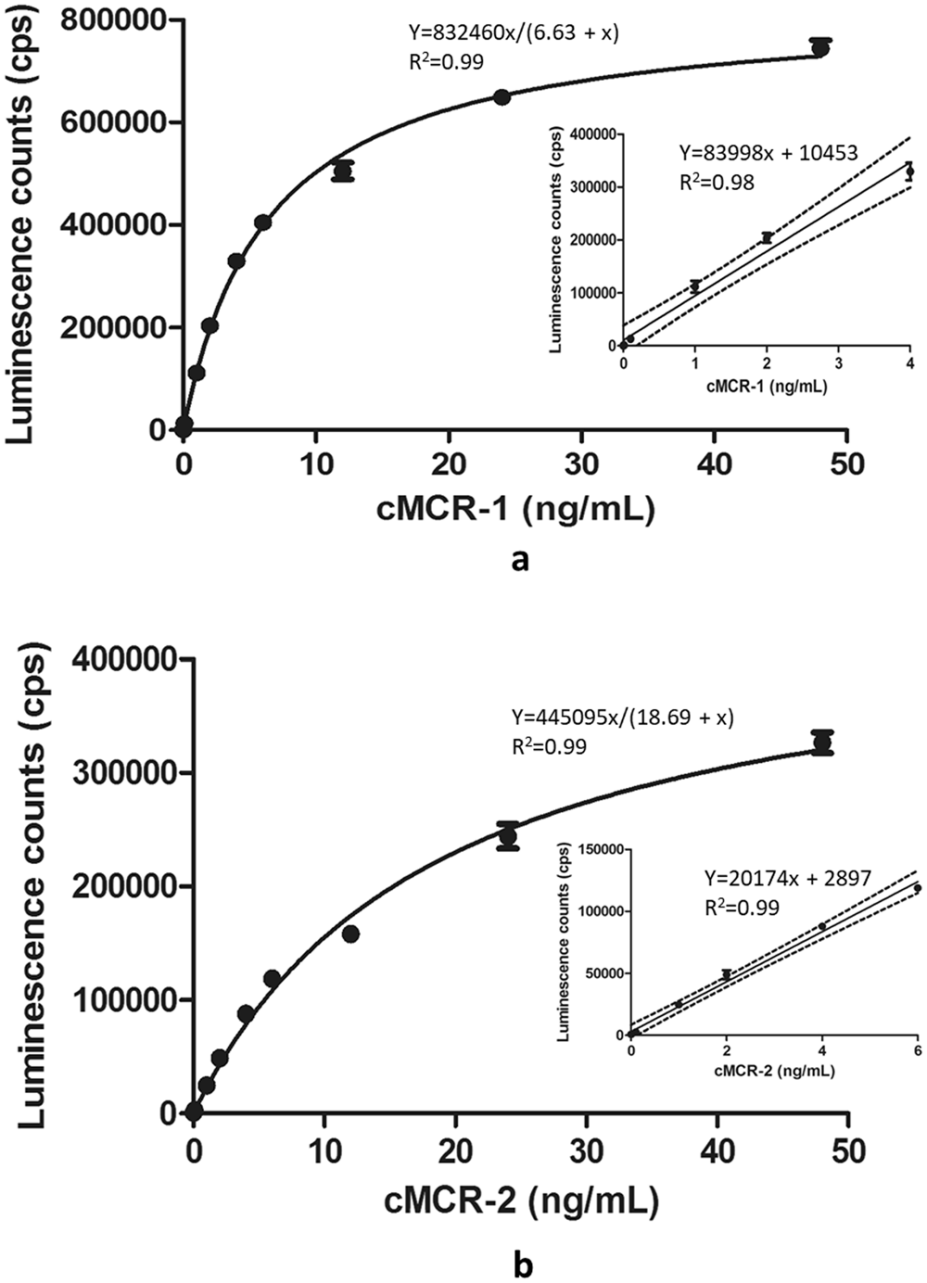
Standard curves for ELISA detection of cMCR-1 (**a**) and cMCR-2 (**b**) in PBS. Luminescence counts (cps) were plotted against the concentration of MCR-1 and MCR-2 proteins (ng/mL). Data represent means of triplicate samples ± SD. The dashed lines in the inset are the upper and lower 95% confidence limits of the linear regression line.

To develop a sensitive sandwich ELISA for MCR proteins, each mAb was evaluated for its performance as a detection antibody by using the pAb as capture antibody (data not shown). In agreement with results obtained from the direct ELISA and Western blot analysis, mAb MCR-1-7 not only detected both cMCR-1 and cMCR-2, but also generated the highest ELISA signal among all mAbs tested in the sandwich ELISA, therefore, it was selected. Figure 5 depicts the detection of the cMCR-1 and cMCR-2 in PBS within the range of 0–50 ng/mL using the sandwich ELISA based on the pAb-MCR-mAb configuration. Good linearity was observed for cMCR-1 quantitation from 0.01–4 ng/mL (R^2^ = 0.98, Fig. 5a inset); for cMCR-2 quantitation from 0.1–6 ng/mL (R^2^ = 0.99, Fig. 5b inset). The average background signal was 563 counts per second (cps) with a standard deviation (SD) of 86.2 for the cMCR-1 ELISA and 833 cps, SD 51.32 for the cMCR-2 ELISA. The limit of detection (LOD) for the cMCR-1 was 0.01 ng/mL and 0.1 ng/mL for the cMCR-2. The ELISA LOD for the cMCR-1 was 10-fold lower than that for the cMCR-2, but the signal to noise ratio for the cMCR-1 was significantly higher than that for the cMCR-2 at all concentrations tested, indicating that this ELISA is more sensitive for detecting the cMCR-1 than the cMCR-2. This is not surprising because both the pAb and the mAb were developed using the cMCR-1 as an antigen. This ELISA was built as one of the most common and affordable sandwich ELISAs, it has a great potential to be improved to meet the requirement needed for various applications by incorporating different signal generation mechanisms or assay platforms^34–36^. To evaluate the precision of the sandwich ELISA, the repeatability (intra-day) and reproducibility (inter-day) of the assay for cMCR-1 and cMCR-2 were evaluated. The intra-assay precision expressed as % coefficient of variation (CV) was less than 5% and the inter-assay precision (% CV) was less than 15% at concentrations within the linear ranges. FDA Center for Biologics Evaluation and Research (CBER) recommends the intra-assay precision determined at each concentration level should not exceed 10% of the CV and the inter-assay precision should not exceed 15% of the CV^37^. The data obtained in this study indicate that the MCR-ELISA is a reliable assay.

### Detection of *mcr*-1 positive bacteria in ground chicken, pork, and beef

**Figure 6:**
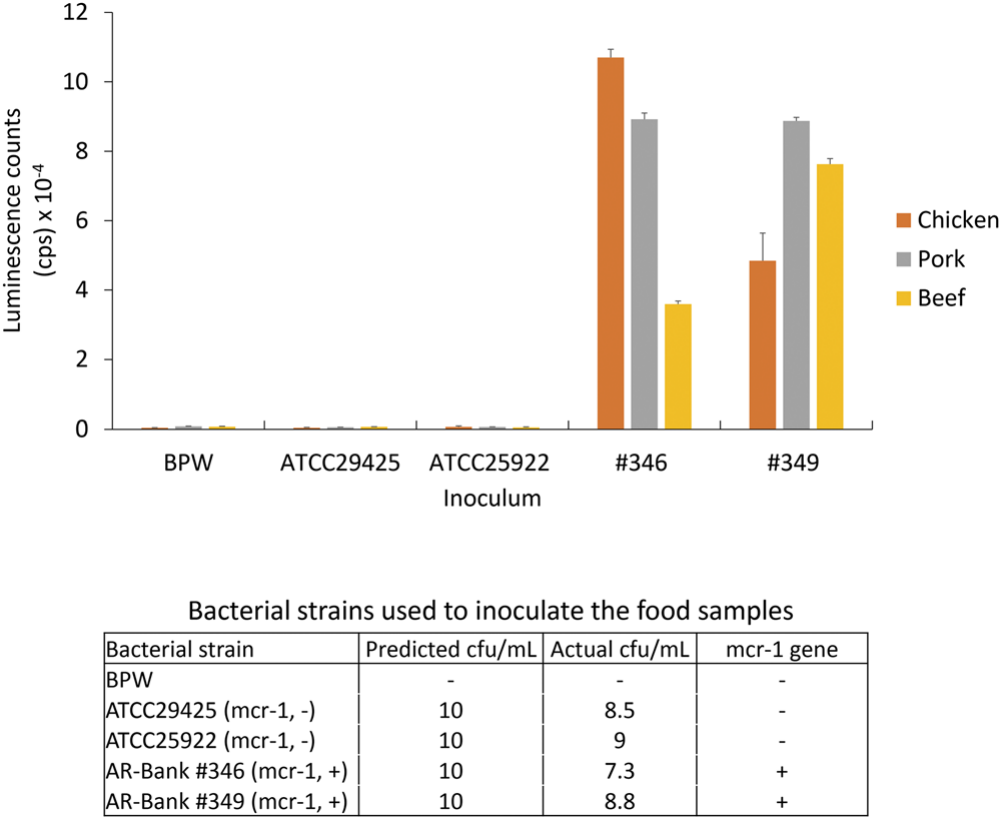
Detection of MCR-1 in ground beef, chicken, and pork samples spiked with bacteria. Negative control (BPW) and bacterial strains were inoculated to meat samples (~10 cfu/25 g) and incubated overnight at 37 °C in Stomacher bags containing 75 mL of TSB with 2 µg/mL colistin. After incubation, 1 mL of sample aliquots were removed from the Stomacher bags. Cells were collected and lysed. A portion of lysate was analyzed by ELISA (100 µL/well).

Plasmid-mediated colistin resistance originated in bacteria associated with food animals^4^ and has been found in bacteria isolated from various types of meat worldwide^6^, threatening human health and safety. Rapid methods for identifying *mcr*-containing strains in food animals are essential. We estimated the sensitivity of the ELISA for detection of MCR-1 produced by bacteria using bacterial culture. Table 2 demonstrates that the ELISA results were positive (above LOD: 227 + 23 × 3 = 296) when cell density was ≥0.4 × 10^6^ for the AR-Bank #346 strain, and ≥4 × 10^6^ cfu/mL for the AR-Bank #349 strain. ELISA results for the *mcr*-1 negative strain, ATCC25922, were negative (below the LOD), at all cell densities tested, even at 8 × 10^8^ cfu/mL, suggesting this ELISA is specific, and does not give false positive results. To validate the ELISA developed in this study for detection of food contamination with the plasmid-borne colistin-resistant bacteria using MCR-1 as a marker, food samples including ground beef, chicken, and pork were spiked with *mcr*-1 positive and negative strains (10 cfu/25 g). The average luminescence counts (cps) obtained from the negative control, Bacterial Protein Extraction Reagent (B-PER), was 667 ± 80 (n = 9). The average counts from the positive control (50 ng/mL cMCR-1 in B-PER) was 751117 ± 15494 (n = 9). As shown in Fig. 6, the ELISA counts for beef, chicken, and pork samples inoculated with Bacto Peptone Water (BPW, Becton Dickinson Company, Sparks, MD), ATCC25922, and ATCC29425 were all below the LOD (907 cps). However, significant amounts of MCR-1 were detected in samples inoculated with the *mcr*-1 positive strains. The luminescence counts measured from the chicken were 106983 ± 2403 and 48447 ± 7981 for strains AR-Bank #346 and AR-Bank #349, respectively; 89230 ± 1784 and 88727 ± 1036 from the pork; 35940 ± 897 and 76263 ± 1628 from the beef. Compared with ELISA signals obtained for water samples inoculated with AR-Bank #346 (805543 ± 6315) and AR-Bank #349 (679970 ± 6163), the signals obtained from the meat samples were relatively low (7.12 to 13.28% of the signals from the water samples), suggesting there were matrix effects associated with these samples. However, this ELISA was still sufficiently sensitive to identify colistin-resistant bacteria inoculated in these complex meat samples, unambiguously. It is worth to note that the results demonstrated in this study was obtained from *mcr*-1 positive strains, as other *mcr*-gene positive strains become available, similar validation assays should be performed.

**Table 2:** LOD for MCR-1 produced by E. coli using the MCR-1 ELISA.

Bacterial strain	*mcr*-*1* gene	cfu/mL	Average counts	SD
AR-Bank #0346	+	4 × 10^5^	407	64
	+	4 × 10^6^	887	107
	+	4 × 10^7^	10980	1164
	+	4 × 10^8^	107503	3039
	+	8 × 10^8^	555867	4447
AR-Bank #0349	+	4 × 10^5^	283	121
	+	4 × 10^6^	490	51
	+	4 × 10^7^	6693	387
	+	4 × 10^8^	71373	679
	+	8 × 10^8^	457703	23821
ATCC25922	−	4 × 10^5^	170	17
	−	4 × 10^6^	247	23
	−	4 × 10^7^	183	40
	−	4 × 10^8^	263	6
	−	8 × 10^8^	267	55
(−) control (LB:B-PER 1:1)	−	0	227	23

## Conclusions

The recent emergence of mobile colistin-resistance in bacteria is of extreme concern as colistin is one of the last line antibiotics effective for severe infections caused by multidrug resistant bacteria. Current methods for colistin susceptibility testing are still fraught with pitfalls. In this study, standards, high affinity polyclonal and monoclonal antibodies against MCR-1 and MCR-2 were developed for the first time. These reagents should be valuable for building various assay platforms for MCR-1 and MCR-2. A sandwich ELISA established using selected antibodies was shown to offer a unique combination of sensitivity and specificity that allowed detection of MCR-1, 0.01 ng/mL in PBS and 0.4 × 10^6^ cfu/mL in cell culture. To simulate real-life assay performance, the ELISA was validated with food spiked with bacteria before culture enrichment and sample preparation. The assay displays great sample tolerance to various meats and the LOD for *mcr*-1-containing bacteria was less than 0.4 cfu/g of ground beef, chicken and pork. The simplicity and reliability of this ELISA makes it a good addition to the current protocols for unambiguous identification of colistin-resistant bacteria in real life samples and it’s feasible to use MCR-1 as an indicator for the presence of viable *mcr*-1-containing bacteria.

## Materials and Methods

### Ethics statement

All procedures with animals were carried out according to institutional guidelines for husbandry (USDA ARS Directive #130.4. v.3) and specific procedures and protocols for antibody production were reviewed and approved by the Western Regional Research Center (WRRC) Institutional Animal Care and Use Committee (IACUC). Mice were euthanized using rapid cervical dislocation.

### Construction, expression and purification of MCR-1 and MCR-2 recombinant proteins

Genes coding for the full-length MCR-1 (residues 1–541 amino acids) and the catalytic domain of MCR-1 (cMCR-1: residues 214–541) were amplified by PCR using the plasmid pBSKSII-KanR-*mcr*1 (kindly provided by Dr. Timothy Plazkill, Baylor College of Medicine, Houston, TX77030) as a template and forward primers 5′-TACTTCCAATCCAATGCAcagcatacttctgtgt-3′ (for the full-length *mcr*-*1* gene), 5′-TACTTCCAATCCAATGCAagtgcgccaaaagatacca-3′ (for the catalytic domain) and reverse primer 5′-TTATCCACTTCCAATGTTATTAgcggatgaatgcg-3′. Genes coding for the full-length MCR-2 (residues 1–538 amino acids) and the catalytic domain of MCR-2 (cMCR-2: residues 212–538) were amplified by PCR using the *mcr*-2 gene synthesized by IDT (www.idtdna.com) based on the published GenBank sequence (NG_051171.1) as a template and forward primers 5′-TACTTCCAATCCAATGCAacatcacatcactcttggtatcgctattc-3′ (for the full-length *mcr*-2 gene), 5′-TACTTCCAATCCAATGCAactgcgccaacagacaccatctatca-3′ (for the catalytic domain) and reverse primer 5′-TTATCCACTTCCAATGTTATTActggataaatgccgcgcggtctttgacctt-3′. PCR products were inserted into the pET His6 TEV LIC cloning vector (1B) (Addgene plasmid #29653). All four MCR-1 and MCR-2 constructs included an N terminal His-tag and were expressed in *E*. *coli* BL21DE3 cells. An overnight culture (20 mL) was used to inoculate 1 L of LB medium supplemented with 50 µg/mL of kanamycin. The cell culture was then incubated at 37 °C with shaking until it reached an OD_600_ of 0.6; at this point, protein production was induced by addition of IPTG at a final concentration of 1 mM. The culture was then incubated at 16 °C for 20 hours. Afterwards, the cells were harvested by centrifugation at 8000 rpm for 30 min at 4 °C. The cell pellet was resuspended in 20 mL buffer A (300 mM NaCl, 20 mM Tris, 20 mM imidazole, pH8, 10% glycerol), supplemented with protease inhibitors (P8849, Sigma-Aldrich, St. Louis, MO63178) and DNase (10 ng/mL, Sigma-Aldrich, Product: 4716728001). For cells carrying the full-length MCR-1 and MCR-2 proteins, the cell pellet was resuspended in buffer A plus 1% Triton-100. Cells were ruptured by sonication and the cell lysate was centrifuged for 1 hour at 15,000 × *g*. The supernatant was passed through a 0.22 µm filter and loaded onto a 5-mL HisTrap FF Crude column (GE Healthcare, Pittsburgh, PA). The proteins were eluted with buffer A supplemented with 500 mM imidazole, pH8, after the column was washed with five bed volumes of buffer A. cMCR-1 and cMCR-2 were further purified by size-exclusion chromatography (SEC) using Superdex 200 - XK 26/70 column (GE Healthcare). For cMCR-1, the N-terminal His-tag was removed by tobacco etch virus (TEV) protease before gel filtration. Protein concentration was determined by absorbance measurements at 280 nm using an absorbance coefficient (0.1%) of 1.03 for cMCR-1 and 1.12 for cMCR-2 (ProtParam tool: http://www.expasy.org/tools/). Mass spectrometry for verification of the full-length recombinant MCR-1 protein was performed by UC Berkeley, Vincent J. Coates Proteomics/Mass Spectrometry Laboratory (http://qb3.berkeley.edu/pmsl/).

### Bacterial strains

*E*. *coli* strains, AR-Bank #0346 and AR-Bank #0349, harboring a *mcr*-1 gene on a multicopy plasmid^38^ were kindly provided by FDA-CDC Antimicrobial Resistance Bank, Atlanta, GA. ATCC25922 (serotype O6) and ATCC29425 (K12) are *E*. *coli* strains purchased from ATCC and used as negative controls for MCR-1.

### Polyclonal antibody production

Production of MCR-1 polyclonal antibody was performed by Pacific Immunology Corp (Ramona, CA) as described previously^39^. Briefly, two rabbits were used, and each rabbit was injected with 300 μg of cMCR-1 each time at 3-week intervals for a total of four injections. Bleeds were collected, and sera were evaluated for binding to cMCR-1 by direct ELISA with 10 ng/mL of cMCR-1 in phosphate buffered saline (PBS) as the plate-coating antigen. Antibodies were purified from pooled sera using Protein-A affinity column (Pierce, Rockfield, IL). Purified IgG concentration was determined by absorbance measurement at 280 nm using the Eppendorf BioSpectrometer (Eppendorf, Hauppauge, NY) and absorbance coefficient (0.1%) of 1.36.

### Monoclonal antibody production

Monoclonal antibodies (mAbs) were prepared and screened as described previously^40^. Briefly, female Balb/cJ mice (Simonsen Laboratories, Gilroy, CA) were immunized at 2-week intervals by intraperitoneal injection (IP) of 100 µL of recombinant cMCR-1 (50 µg/mL) in Sigma Adjuvant System (Sigma, St. Louis, MO). Following the third injection, sera were obtained (50 µL/mouse) and evaluated for anti-MCR-1 titers. Mice with a strong antibody titer were boosted with a fourth IP injection two week after the third injection with a single dose of cMCR-1 (100 µL at 10 µg/mL in PBS without adjuvant). Three days later, mice were euthanized and their splenocytes were fused with SP2/0 myeloma cells using polyethylene glycol as previously described^41^. The culture media of hybridomas were screened with a direct ELISA using 0.1 µg/mL of cMCR-1 in PBS as the plate-coating antigen. The positive cell cultures were cloned 3–4 times by limiting dilution until selected hybridomas are clonal. Selected hybridomas were then expanded and antibodies were purified from culture supernatant by affinity chromatography using a Protein-G conjugated Sepharose column (Sigma, #P-32196) and bound antibodies were eluted with 0.1 M glycine-HCl, pH 2.7. Purified IgG concentration was determined by absorbance measurement at 280 nm using the Eppendorf BioSpectrometer (Eppendorf, Hauppauge, NY) and absorbance coefficient (0.1%) of 1.36. Antibody isotype was determined by ELISA using cMCR-1 coated microtiter plates and horseradish peroxidase (HRP)-conjugated, isotype-specific antibodies (SouthernBiotech, Birmingham, AL).

### ELISAs

Direct ELISAs were performed by directly coating variable amounts of cMCR-1 in wells of black NUNC plates (Thermo Fisher Scientific, Waltham, MA) overnight at 4 °C. Plates were then blocked with a blocking buffer [BB: 5% non-fat dry milk (NFDM) in 0.02 M Tris-buffered saline with 0.9% NaCl, pH 7.4 and 0.05% Tween-20 (TBST)] for 1 hour at room temperature (RT). Different mAbs (1 µg/mL in BB) were added to the plates and incubated for 1 hour at RT after washing two times with TBST. After washing six times with TBST, the plates were incubated with goat anti-mouse IgG conjugated with HRP (GAM-IgG-HRP, Promega, Madison, WI, 200 ng/mL in BB) for 1 hour at RT. Finally, SuperSignal West Pico Chemiluminescent Substrate (Pierce) was added and luminescence counts were measured in counts per second (cps) using the Victor-3 plate reader (Perkin-Elmer, Shelton, CT). The procedures and reagents used for the sandwich ELISAs were similar to the direct ELISA described except that the plates were first coated with the rabbit pAb against the cMCR-1 (1 µg/mL in PBS), and then the MCR-1 proteins were added. The detection antibody was mAb MCR-1-7 (100 ng/mL in BB). ELISA limit of detection (LOD) was determined using the lowest concentration of analyte that generated a response greater than the background plus three times the standard deviation. For each ELISA test, three independent experiments in triplicate were performed and one representative data set is presented. Statistical significance was determined by two-tailed unpaired Student’s *t*-test (*p* < 0.05 was considered significant). ELISA standard curves were generated using GraphPad Prism 6 (GraphPad Software, La Jolla, CA). ELISA intra-day precision was determined based on results obtained from two independent assay runs within a day. Three replicates were analyzed for each concentration at each run. The % CV for each concentration was calculated by dividing the standard deviation (SD) by mean ELISA readings calculated from two runs (total 6 replicates) and multiplying by 100. The inter-day precision (% CV) was determined by dividing the SD of 3 day means by mean of 3 day means, and multiplying by 100.

### Polyacrylamide gel electrophoresis (PAGE) and Western blot

All gel electrophoresis equipment, buffers, gels and PVDF membranes were purchased from Invitrogen (Thermo Fisher Scientific). MCR-specificity of each mAb was analyzed by Western blot. Protein samples were separated by SDS-PAGE using 4–12% NuPAGE (denatured) Novex Bis-Tris mini gels following the manufacturer’s protocol. To visualize proteins directly after gel electrophoresis, 2 µg of proteins were loaded in each lane and gels were stained with SimplyBlue SafeStain (Thermo Fisher Scientific). For Western blot analysis, 0.2–0.5 µg/lane of proteins were loaded and separated by SDS-PAGE. Proteins were electronically transferred to PVDF membranes (0.45 um). The membranes were blocked with 5% NFDM, then probed with mAbs (500 ng/mL), followed by GAM-IgG-HRP (25 ng/mL). Bound antibody was detected using the Amersham ECL-Plus Western Blotting Detection System (GE Healthcare, UK) according to manufacturer’s protocol.

### Detection of MCR-1 in meat samples inoculated with bacteria

Meat samples, including ground beef, chicken, and pork were purchased from a local supermarket and packed into stomacher bags (Seward Stomacher 400 Classic Filter Bags, Nelson Jameson Inc., Marshfield, WI), 25 g/bag. Samples were kept at 4 °C before use. Inocula of *E*. *coli* strains were created by serially diluting an overnight axenic culture from frozen stock, shaken and grown at 37 °C, into buffered peptone water (BPW), to a desired cfu/mL of 10 cfu per 25 g (or mL) of sample. Actual inoculum levels were later determined via spread-plating 0.1 mL of cultures onto tryptic soy agar (TSA) plates and incubating overnight at 37 °C. One mL of the prepared dilutions or BPW control was added to the samples. Enrichment broth, 75 mL of tryptic soy broth (TSB) with colistin (2 µg/mL), was then added to dilute 25 g (or mL) of each sample, and the sample-broth solution was mixed by hand-massaging. Enrichment was then performed in a growth chamber for 16 hours at 37 °C with shaking (100 rpm). Post enrichment, 1 mL sample aliquots were removed from the Stomacher bags and pipetted into microcentrifuge tubes. After centrifugation, bacterial cell pellets were lysed with 1 mL B-PER containing protease inhibitors (P8849, Sigma-Aldrich) and DNase (10 ng/mL, Sigma-Aldrich, Product: 4716728001). A portion (100 µL) of each sample aliquot was added to the ELISA microtiter wells for ELISA test.

## Electronic supplementary material

Supplementary Information
