# Supplementary material for: Development of novel antibodies for detection of mobile colistin-resistant bacteria contaminated in meats
Source: Sci Rep. 2018 Nov 13;8:16744. doi: 10.1038/s41598-018-34764-2 (PMC6233175; doi:10.1038/s41598-018-34764-2)
Supplement: Supplementary file 1 — Supplementary Information [file 41598_2018_34764_MOESM1_ESM.pdf]

Development of novel antibodies for detection of mobile colistin-resistant bacteria contaminated in meats

Xiaohua He[1,*], Daniela Mavrici[1, 2], Stephanie Patfield[1], and Fernando M. Rubio[3]

[1]Western Regional Research Center, U.S. Department of Agriculture, Agricultural Research Service, Albany, 94710, USA
[2]Current address: Plexxikon Inc., 91 Bolivar Drive, Berkeley, 94710, USA
[3]Abraxis, Inc., Warminster, 18974, USA

*Correspondence and requests for materials should be addressed to X.H. (email: xiaohua.he@ars.usda.gov)

Supplementary Fig. S1

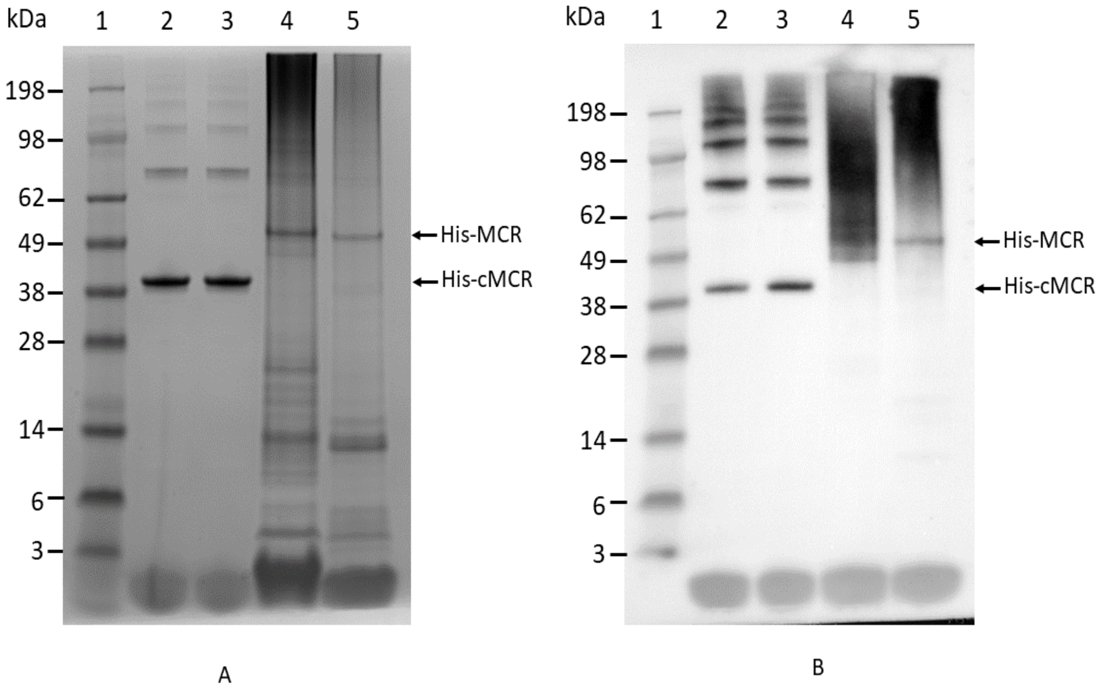

Figure S1. Analysis of His-tagged recombinant MCR-1 and MCR-2 proteins.

A. Full-length SDS-PAGE of partially purified MCR recombinant proteins stained with SimplyBlue: lane 1, Protein markers with molecular weights (kDa) indicated at the left; lane 2, cMCR-1; lane 3, cMCR-2; lane 4, MCR-1; lane 5, MCR-2.

B. Full-length Western blot of partially purified MCR recombinant proteins using anti-His antibody: lane 1, Protein markers with molecular weights (kDa) indicated at the left; lane 2, cMCR-1; lane 3, cMCR-2; lane 4, MCR-1; lane 5, MCR-2. The predicted His-MCR (full-length) and His-cMCR (catalytic domain) protein bands are indicated by arrows at the right.

Supplementary Fig. S2

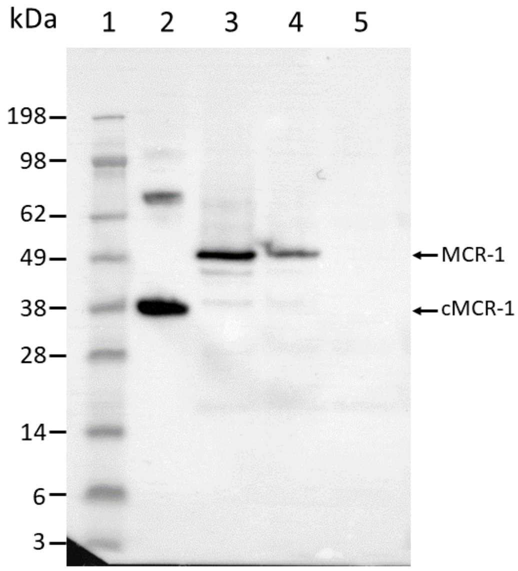

B

Figure S2. Activity of immunized rabbit serum to MCR-1.

B. Full-length Western blot of MCR-1 produced by bacterial cells using immunized rabbit serum IgG. Lane 1, Protein markers with molecular weights (kDa) indicated at the left; Lane 2, Purified cMCR-1 (0.5 µg); Lanes 3 to 5, Cell lysates from bacterial strains AR-Bank #346, AR-Bank #349, and ATCC25922 (colistin negative strain) respectively. Samples were separated by SDS-PAGE under non-reducing condition. The expected sizes of the recombinant MCR-1 catalytic domain and full-length MCR-1 produced by wild type bacteria are indicated at the right side of the blot.

Supplementary Fig. S3

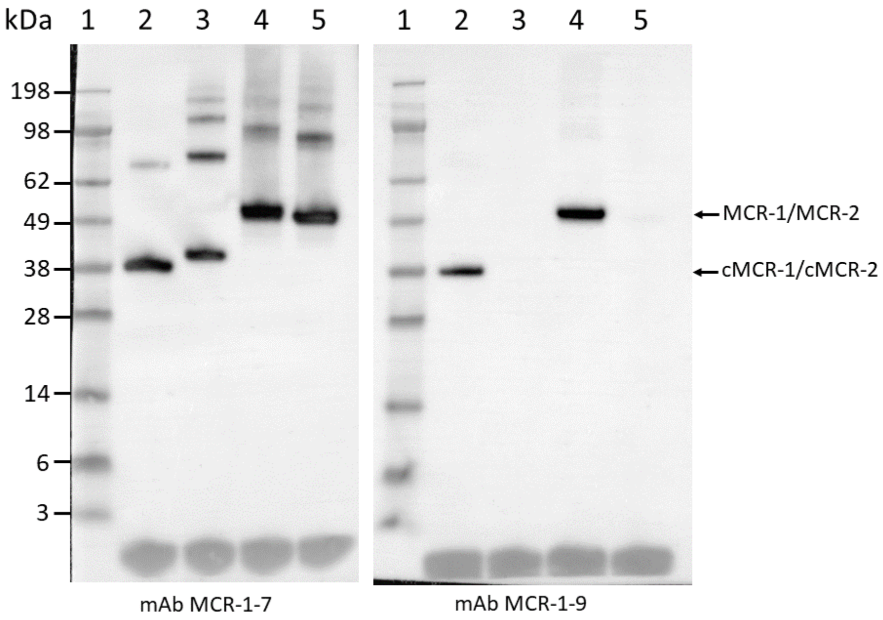

Figure S3. Full-length Western blot of mAb reactivity to MCR-1 and MCR-2. Lane 1, Protein markers with molecular weights (kDa) indicated at the left; Lane 2, Purified cMCR-1 with His-tag removed (0.2 µg); Lane 3, Purified cMCR-2 with His-tag on (0.2 µg); Lanes 4 and 5, Purified His-full-length MCR-1 and His-full-length MCR-2. Blots were probed with indicated mAbs following SDS-PAGE under non-reducing condition. The expected sizes MCR-1 and MCR-2 proteins are indicated at the right side of the blot.
